# Smartphone Application with Virtual Reality Goggles for the Reliable and Valid Measurement of Active Craniocervical Range of Motion

**DOI:** 10.3390/diagnostics9030071

**Published:** 2019-07-10

**Authors:** Ke-Vin Chang, Wei-Ting Wu, Mei-Chu Chen, Yi-Chi Chiu, Der-Sheng Han, Chih-Cheng Chen

**Affiliations:** 1 Department of Physical Medicine and Rehabilitation, National Taiwan University Hospital, Bei-Hu Branch 10845, Taiwan; 2 Community and Geriatric Medicine Research Center, National Taiwan University Hospital, Bei-Hu Branch 10845, Taiwan; 3 Department of Physical Medicine and Rehabilitation, National Taiwan University College of Medicine, Taipei 10048, Taiwan; 4 Health Science and Wellness Center, National Taiwan University, Taipei 10617, Taiwan; 5 Institute of Biomedical Sciences, Academia Sinica, Taipei 11574, Taiwan; 6 Taiwan Mouse Clinic—National Comprehensive Mouse Phenotyping and Drug Testing Center, Academia Sinica, Taipei 11574, Taiwan

**Keywords:** goniometer, neck, rehabilitation

## Abstract

Objective: This study aimed to determine the intra-rater and inter-rater reliability and validity of a hybrid device, combining virtual reality goggles, a magnetometer and an inclinometer application for smartphones, to measure craniocervical range. Summary of Background Data: Accurate evaluation of craniocervical range of motion is important for early detection of certain diseased conditions and monitoring the progress of interventions. The universal goniometer is widely used for the measurement but it requires experienced practitioners. Whether a combination of virtual reality goggles and smartphone applications can provide the same or better performance compared with the goniometer is still unknown. Methods: Forty-one healthy adults from the department of physical medicine and rehabilitation were recruited for craniocervical range examination (flexion, extension, side-bending to the right or left and rotating to the right or left) by using the hybrid device and universal goniometer. Using the hybrid device, repeated measurements were performed twice by a primary rater and once by a second rater. The primary rater also conducted a measurement using the universal goniometer in the same cohort. The intra-rater and inter-rater reliability (intra-class correlation coefficient (ICC)) were calculated using the two-way random effect model, whereas the validity was examined by the Pearson correlation coefficient and Bland-and-Altman plot. The interval between the first and second sessions of the measurement for intra-rater reliability was set at 30 min. Results: Excellent intra-rater (ICC ≥ 0.925) and inter-rater (ICC ≥ 0.880) reliability was noted for the hybrid device. The minimal detectable changes from intra-observer and inter-observer comparisons ranged between 4.12° and 7.42° in all six directions. The Bland-and-Altman plot revealed small mean differences (≤1.68°) between the hybrid device and universal goniometer. Both instruments had highly correlated measurements of craniocervical motion (*r* values ≥ 0.918). Conclusion: For healthy participants, excellent intra-rater and inter-rater reliability was noted for the hybrid device, and the measurements were consistent with the universal goniometer measurements. Future studies are needed to examine whether the device can perform similarly for patients with neck disorders.

## 1. Introduction

Neck pain is a common musculoskeletal complaint, with a mean prevalence of 23.1% in the general population [1]. In addition to pain, these patients may present with weakness, numbness and limitation of range of motion. Accurate evaluation of craniocervical range of motion is a crucial part of physical examinations for neck disorders. Limited craniocervical motion may result from myofascial pain syndrome, different kinds of arthritis affecting cervical spines, and acute/chronic paraspinal muscle strain following traumas [2,3]. Furthermore, cases with restricted movement of head and neck are associated with a higher risk of repeated injury [4]. Therefore, precise measurements of craniocervical range might be helpful for early detection of certain diseased conditions, like atlantoaxial instability or subaxial subluxation in rheumatoid arthritis [5] and monitoring the progress of interventions.

The universal goniometer is widely used for the measurement of the range of motion of joints [6,7], mainly due to ease of use, portability and low cost. The goniometer-based approach provides reliable and valid measurements of cervical motion in an asymptomatic population [8] or patients with spine disorders, such as ankylosing spondylitis [9]. Another commonly used tool is the inclinometer, also showing its satisfactory reproducibility and validity in evaluations of neck range of motion in participants with [10] and without neck pain [11]. Compared with the goniometer, the inclinometer is likely to have better reliability and agreement in terms of measuring cervical range [7] However, a reliable assessment by using both instruments requires experienced medical practitioners who are familiar with the examination procedure, as well as body surface landmarks. Taking neck side-banding as an example, the examiner needs to reposition the participant’s neck to the vertical axis and correctly place the center of the goniometer on the C7 spinous process before starting. In challenging cases with conditions like scoliosis and short neck, the inexperienced investigator may have difficulty finding the neutral position of the neck or locating the bony prominence of C7. In addition to the experience and training of the examiners, digitization of the assessment tools [11] and direction of cervical motions also play a crucial role in reliability and validity of measurements [12].

Recently, a number of applications have been developed to simulate goniometers or inclinometers, so as to allow convenient measurement of joint range using smartphones [13,14]. Those iPhone [15] or Android smartphone [16] based applications also demonstrate their usefulness in the assessment of cervical range of motion. Until now, there have not been many studies comparing the performance of such applications with the universal goniometer regarding craniocervical range. We have developed a device combining virtual reality goggles, a magnetometer and an inclinometer on a smartphone. This study aimed to examine the reliability and validity of the hybrid equipment to measure craniocervical range in a healthy population. We hypothesized that this new tool would have similar or even better reliability and validity compared with the goniometer.

## 2. Materials and Methods

### 2.1. Participants

This was a study of clinical measurement (reliability and validity) using a two-stage repeated measures design. The study was conducted in a community hospital in East Asia. All the participants were healthy adults with no reported major systemic disorders. Based on the regulation of our hospital’s institutional review board, the lowest age limit for adults was 20 years. As we intended to apply this device to different age groups in the future, no upper age limit was set. Those who complained of any grade of neck pain were excluded. We also excluded people who received physical therapy or injections to neck region in the last 6 months or any type of surgeries in the neck and shoulder regions. All participants were required to submit informed consent prior to the study, which was approved by our hospital’s institutional review board (201509022RINB, 14 October 2015 approved).

### 2.2. Hybrid Device for Evaluation of Cervical Range of Motion

**Figure 1:**
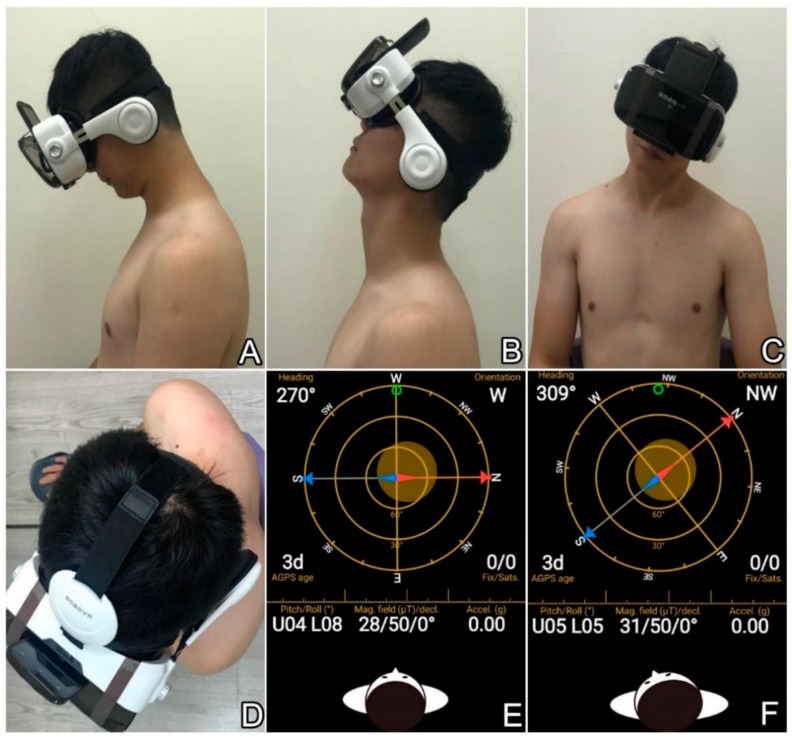
Use of the hybrid device to measure craniocervical ranges in (**A**) flexion, (**B**) extension, (**C**) side-bending and (**D**) rotating. Illustration of how the rotation angle is measured: (**E**) the heading in the neutral position shows 270°; (**F**) the heading when the head was maximally rotated to the left shows 309°. Therefore, the rotation angle would be 39°.

The application software, GPS status and tool box version 8.4.177 (Hungary, 1033 Budapest, Hévízi u. 5.), was installed into a smartphone equipped with a gyroscope and a magnetometer. The smartphone was then fitted to virtual reality goggles. The screen of the phone faced toward the examiner’s body to prevent the patient from being aware of their motion magnitude. What the participant visualized in the virtual reality goggles was only the reflection from a piece of black glass. During examination, the participants were seated with their torso upright against the back of the chair. Their arms were naturally hung beside the body with the hips, knees and ankles all at 90° of flexion. After wearing the goggles, the participants were asked to maximally flex their neck toward the chin and anterior chest (Figure 1A). Then they elevated the chin and extended the neck to the most tolerable range (Figure 1B). Next, they proceeded to maximal neck side-bending in both directions along the coronal plane (Figure 1C). Lastly, they were required to position their neck and back to the upright posture for examination of the maximal angle of neck rotation (Figure 1D), without shrugging their shoulders. The participants performed all the tests three consecutive times and the maximal values of neck movement in each direction were used for analysis. Before each test, the examiner captured a snapshot of the screen to obtain the basal magnetometer and inclinometer status without viewing the data, as the displayed part was inside the goggles (Figure 1E). Likewise, another snapshot was taken when the participant was positioned at the maximal range of the required motion (Figure 1F). The range of craniocervical motion was acquired by calculating the difference in the angle of the target axis between different positions. Because the participants might have neck muscle fatigue after wearing the goggles for a long period, the measurement with the virtual reality goggles was conducted separately from that with the goniometer. In addition, the virtual reality goggles only served as a device on which the smartphone could be mounted. There was no virtual reality technology used in the present study.

### 2.3. Universal Goniometer for Assessment of Cervical Range of Motion

**Figure 2:**
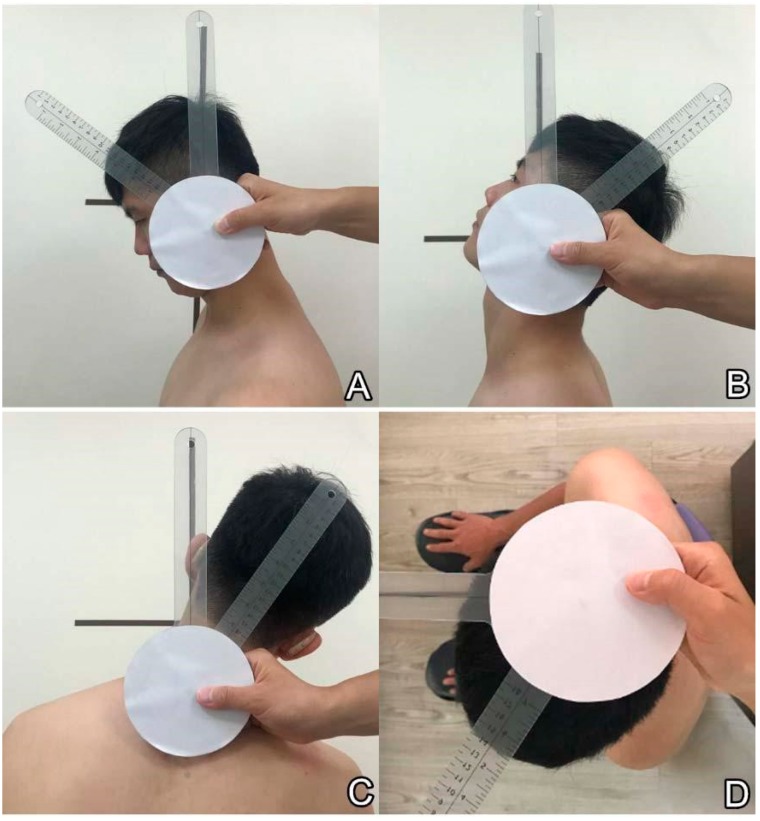
Use of the universal goniometer to measure craniocervical ranges in (**A**) flexion, (**B**) extension, (**C**) side-bending and (**D**) rotating. During the examination, some vertical and horizontal lines were marked on the wall and floor as references for cervical motions and goniometer placement. One side of the scale was covered by white paper. The investigators were not aware of the exact angles until the end of examination when the non-covered side is flipped toward them.

The participants were seated with their head in the neutral position for the assessment of cervical range using the universal goniometer [6]. During the examination, vertical and horizontal lines were marked on the wall and floor as references for cervical motions and goniometer placement. One side of the scale was covered by a white paper to ensure the investigators were not aware of the exact measurements until the end of examination. The goniometer axis was placed on top of the mastoid process along the sagittal plane to test the angle of neck flexion (Figure 2A) and extension (Figure 2B). To measure the range of neck side-bending to the right or left, the goniometer axis was positioned on the spinous process of the seventh cervical vertebrae (Figure 2C). The goniometer axis was on the center of the head during the examination of the extent of neck rotation to the right or left (Figure 2D). All the examinations were performed three times and the maximal values were used for analysis. Furthermore, because the reproducibility of the goniometer measurement for cervical range of motion has been well established by previous research [7], its intra-rater and inter-rater reliability was not examined in this study.

### 2.4. Flow of Measurement of Craniocervical Range

All participants were evaluated independently by both raters by using the hybrid device for the first session. Both raters were selected randomly from the board-certificated physical therapists (more than 1 year of working experience) in the department of physical medicine and rehabilitation in our hospital. At the first session, the order of the raters was randomized. The random sequence was generated by using the statistical analytic software, MedCalc (version 14, Ostend, Belgium). After half an hour, the main rater conducted a repeated measurement employing the hybrid device. Another half an hour later, the main rater measured range of motion again by using the universal goniometer. The measurement with the hybrid device always preceded that with the goniometer.

### 2.5. Sample Size Estimation

We estimated the required sample size by using the correlation coefficient. We assumed that the measurements between the hybrid device and universal goniometer had an *r* value of 0.5 based on previous similar studies [17,18]. The type I and II errors were set below 0.05 and 0.2, respectively, and the attrition of the participants was allowed to be 20%. The total number needed was at least 37 people.

### 2.6. Statistical Analysis

The intra-rater reliability was calculated from the repeated measurements of the first rater using the hybrid device, whereas the inter-observer reliability was derived from the first hybrid device evaluation of each observer. The data were analyzed by the two-way random model and were expressed by the intraclass correlation coefficient (ICC) using the two-way random effect model and its 95% confidence interval (CI) [19]. The standard error of measurement (SEM) was calculated using the following formula: the standard deviation pooled from both evaluations × square root (1 − ICC) [20]. The minimal detectable change (MDC) was obtained employing the following equation: MDC = 1.96 × √2 × SEM [20].

A Bland–Altman plot was used to demonstrate the construct validity between the hybrid device and universal goniometers [21]. The plot was also used to investigate an association of the differences with the magnitude of measurements, to detect potential systemic bias, and to recognize outliers. The *x* and *y* axes indicated the mean of the paired set of measurements and its between-group difference, respectively. The limits of agreement (LoA) were defined as the mean difference ±1.96 standard deviations of differences. The coefficient of repeatability (COR), equaling to 1.96 standard deviations of between-group differences, was used to represent the precision of the hybrid device measurement in contrast to the universal goniometer measurement. The paired t-test compared the mean differences between two evaluations, and Pearson correlation analysis was also performed for data obtained by both methods. All the analyses were conducted by using MedCalc (version 14, Ostend, Belgium) and a *p* value < 0.05 was considered statistically significant.

## 3. Results

The present study consisted of 41 participants, 78% of whom (*n* = 32) were female. Their average age was 36.9 years with a standard deviation of 7.6 years. Using the hybrid device, a significant difference was detected between repeated evaluations of the same raters in side-bending to the right (mean difference: 0.75°, *p* = 0.038) and rotating to the left (mean difference: 1.07°, *p* = 0.019) (Table 1). The intra-rater reliability (ICC) ranged from 0.925 (95% CI, 0.864 to 0.959) for side-bending to the right to 0.963 (95% CI, 0.933 to 0.980) for flexion. The intra-rater SEM and MDC ranged from 1.48 to 2.68° and from 4.12 to 7.42°, respectively (Table 2).

When comparing measurements by different users, there was a significant difference in inter-rater reliability between the evaluations of both observers in side-bending to the left (mean difference: 0.97°, *p* = 0.008) (Table 1). The ICC ranged between 0.880 (95% CI, 0.786 to 0.934) for side-bending to the right and 0.979 (95% CI, 0.962 to 0.989) for flexion. The inter-rater SEM and MDC ranged from 1.59 to 2.61° and from 4.39 to 7.24°, respectively (Table 2).

**Figure 3:**
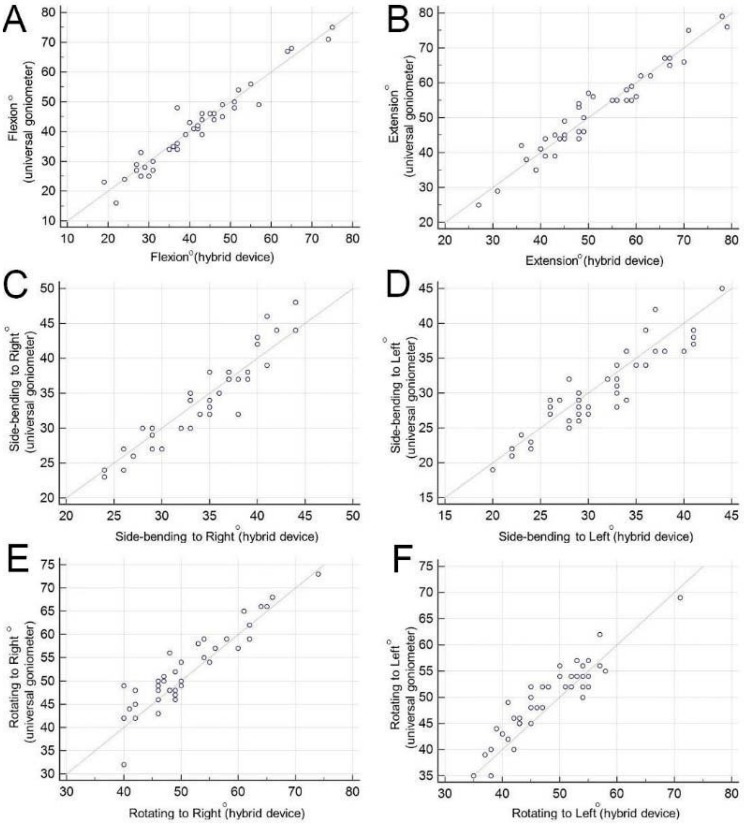
The correlation of craniocervical ranges measured by the hybrid device and universal goniometers in (**A**) flexion, (**B**) extension, (**C**) side-bending to right, (**D**) side-bending to left, (**E**) rotating to right and (**F**) rotating to left.

**Figure 4:**
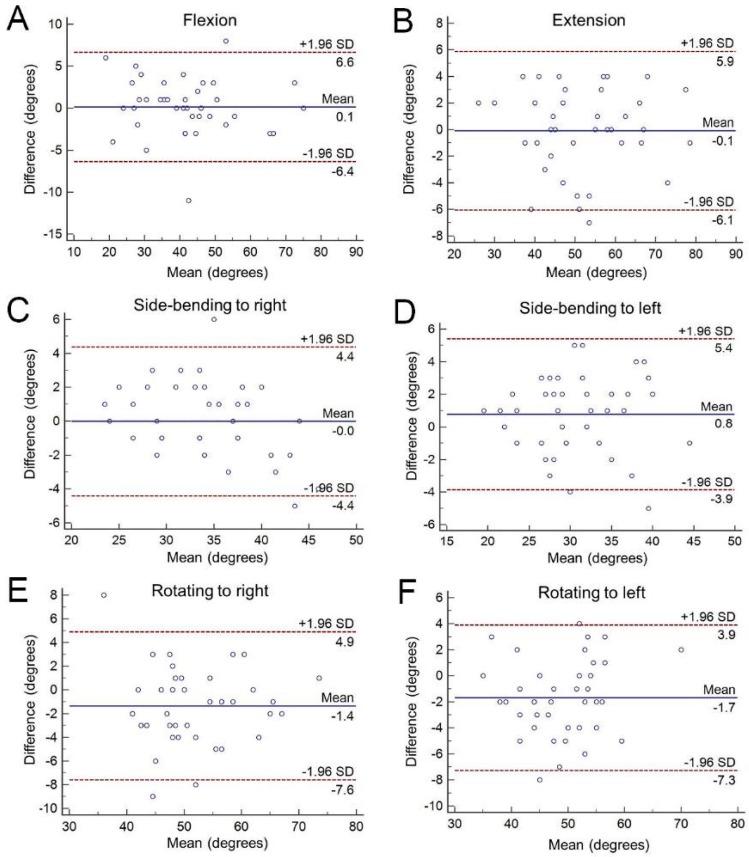
The Bland–Altman plot for craniocervical ranges measured by the hybrid device and universal goniometers in (**A**) flexion, (**B**) extension, (**C**) side-bending to right, (**D**) side-bending to left, (**E**) rotating to right and (**F**) rotating to left. The blue solid line indicates the mean values of all he subjects, whereas the red dotted line refers to its upper and lower limits (mean ± 1.96 standard deviation).

The measurements obtained by the hybrid device correlated well with the measurements of the universal goniometer (*r* values ≥0.918) (Table 3, Figure 3). Significant difference between the methods was observed in side-bending to the left (mean difference: 0.75, *p* = 0.047), rotating to the right (mean difference: 1.36, *p* = 0.009) and rotating to the left (mean difference: 1.68, *p* < 0.001) (Table 3). The Bland–Altman plots suggest that only two of 41 subjects for flexion, one of 41 subjects for extension, two of 41 subjects for side-bending to the right, one of 41 subjects for side-bending to the left, two of 41 subjects for rotating to the right and one of 41 subjects for rotating to the left lie beyond the 95% confidence intervals (Figure 4). These data indicate agreement between both measuring systems for various directions of neck range of motion. The coefficient of repeatability ranged from 5.888° to 6.720° for measurement of neck movement in all directions (Table 3). Regarding a few outliners in which there was a discrepancy between the hybrid device and goniometer, we did not find any unusual points on their body weights and heights, which were within 2 standard deviations of the means of all participants.

## 4. Discussion

To the best of our knowledge, the present study is the first to examine the reliability and validity of a smartphone application combined with virtual reality goggles in measurements of craniocervical range of motion. Our study revealed that the hybrid device provided high intra-rater and inter-rater reliability for measuring craniocervical range of motion. We also demonstrated an excellent agreement between the hybrid device and the universal goniometer, indicating both measurement techniques can be used interchangeably.

In recent years, an increasing number of studies have investigated the usefulness of smartphone applications in measurement of cervical range of motions. Tousignant-Laflamme et al. [15] used an iPhone application to measure cervical movement in 28 healthy participants, showing moderate (ICC > 0.50) to good (ICC > 0.65) criterion validity of directions in flexion, extension, lateral flexions and right rotation using the inclinometer as the standard. Likewise, Quek et al. [16] used an android phone application to evaluate cervical range of motion, demonstrating excellent intra-rater reliability for cervical flexion, extension and lateral flexion (ICC = 0.82–0.90). Stenneberg et al. [22] further applied a similar smartphone application, on patients with neck pain and found that ICCs for concurrent validity and inter-rater reliability were higher than 0.90 Therefore, the inbuilt smartphone inclinometer seems to be useful for evaluation of neck motion.

Compared with assessing peripheral joint motion, craniocervical range is more challenging to measure. Craniocervical motion is three-dimensional but not restricted in a single plane. Movement in one axis can be influenced by that in other dimensions. Additionally, there are multiple joints involved in neck motion, so estimation of craniocervical range may vary significantly when the reference joint is different. Pourahmadi et al. [17] first examined the reliability and contrast validity of the G-pro iPhone application in evaluation of craniocervical motion in patients with neck pain. They found moderate to good intra-rater (ICC ≥ 0.66) and inter-rater (ICC ≥ 0.66) reliability of the application and high correlation with the universal goniometer (*r* values ≥ 0.63). However, in their experiments, the iPhone was not firmly attached to the participants’ bodies and the reciprocal anatomical discrepancy between the examiner’s hand and examinee’s reference landmark might be a main source of variation. Therefore, in our experiments we fit the smartphone onto virtual reality goggles and tested if the reliability and validity were better than the previous experiments using an unsecured device.

In the present research, we found that the hybrid device could reliably provide craniocervical range in six directions, with the ICC within or between the raters ranging from 0.880 to 0.979, which was higher than that reported from Pourahmadi et al. [17]. We speculate that the satisfactory outcome resulted not only from the addition of the goggles to the smartphone application, but also from the standardized protocol of measurements in all six directions. Although there were some significant differences among/between raters in neck side-bending and rotating to the left (Table 3), the average discrepancy was minimal, all less than 2°. The SEMs of neck motion in six dimensions were all within 2.68°, indicating excellent precision of measurement by the hybrid device.

Regarding the consistency between the hybrid device and universal goniometer, the Bland–Altman plot revealed excellent agreement, with a maximum of only two out of each of the 41 paired measurements beyond the 95% confidence interval. There were significant differences between both techniques in side-bending to the left and head rotating to the right and to the left. The measurements for head rotation in the right and left directions differed by more than 1° (1.36 and 1.68°, respectively) (Table 3). Extension and side-bending have reference points for the universal goniometer measurement that are clear surface landmarks (mastoid process and C7 spinous process). However, for neck flexion, the center of the head may be challenging to precisely locate. Additionally, the angle of head rotation also depends on the vertical axis of the occipital-axial joint. Therefore, the motion of head rotation may not exactly take place at the horizontal plane if it is measured by a universal goniometer. These factors are less likely to influence the measurement by the hybrid device, as it used a gyroscope and a magnetometer to estimate the position of the target in three-dimensional space.

Compared with the hybrid device, the medical practitioners are still more familiar with the goniometer and inclinometer. In addition, there are more validation studies conducted with both aforementioned instruments regarding evaluation of cervical ranges of motions [7]. However, there are at least two advantages of using the hybrid device to measure craniocervical range. First, the data shown on the smartphone are digitalized, which can significantly reduce the error from reading the scales on the universal goniometer. Second, we can let the screen of the smartphone fit into the goggles, which allows the participant to report/document their own neck motion without assistance. Therefore, people can employ the hybrid device as a piece of biofeedback equipment and a rehabilitation tool to monitor and improve their own cervical range of motion.

A virtual reality headset is known to cause nausea and imbalance after prolonged use. The weight of the headset may cause or worsen neck pain in patients with cervical spine disorders. Therefore, as the application can be installed on nearly every brand of smartphone using the android system, we would like to suggest that the investigators choose lighter smartphones to reduce the overall weight of the hybrid device. In addition, a sufficient interval, like 5 min, is needed between different sessions of measurements. This can also prevent the participants from having neck muscle fatigue during the examination.

Several limitations need to be acknowledged. First, the accuracy of measurements on the hybrid device depends entirely on the sensors of the smartphone. Although the variability is supposed to be small, calibration could be necessary to make sure the gyroscope and magnetometer function properly. In addition, the examiners are required to check whether the smartphone is firmly secured to the goggles, since any reciprocal movement will affect the measuring precision. Second, the hybrid device was only tested on healthy participants who did not report any cervical complaints in the present study. It remains to be seen if the device can still have such an excellent performance on patients with cervical pain, which should be examined on the future studies. Third, the interval between the first and second sessions of evaluations was not long enough and the result might be biased by the training effect or compensation. Fourth, the reliability of the goniometer is less than for the inclinometer [7]. Through the present study, we were still unable to know whether the hybrid device would have equivalent or better performance than the inclinometer. In addition, the 3D kinematic analysis emerged as the gold standard for cervical range measurement [23], which should be used in our future study to further examine the validity of our hybrid device.

## 5. Conclusions

In our study, we found high intra-rater and inter-rater reliability in measuring craniocervical range using a device incorporating virtual reality goggles and the magnetometer and inclinometer on a smartphone application. In healthy participants, the hybrid device was in excellent agreement with the universal goniometer. Future studies should be conducted to examine if the device can achieve a similar performance on patients with neck disorders.

## Figures and Tables

**Table 1:** Intra-rater and inter-rater evaluations and corresponding differences of craniocervical ranges measured by the hybrid device.

	First Rater First Evaluation in Degrees (95% CI)	First Rater Second Evaluation in Degrees (95% CI)	Second Rater First Evaluation in Degrees (95% CI)	Intra-Rater Mean Difference in Degrees (95% CI)	*p* Value	Inter-Rater Mean Difference in Degrees (95% CI)	*p* Value
Flexion	41.80 (37.70 to 45.90)	41.63 (37.78 to 45.47)	41.46 (37.58 to 45.34)	−0.17 (−1.23 to 0.89)	0.748	−0.34 (−1.14 to 0.46)	0.397
Extension	51.48 (47.56 to 55.40)	50.87 (47.39 to 54.36)	52.09 (48.36 to 55.83)	−0.60 (−1.72 to 0.50)	0.275	0.60 (−0.55 to 1.77)	0.296
Side-bending to right	34.51 (32.78 to 36.23)	33.75 (31.78 to 35.72)	34.09 (32.12 to 36.06)	−0.75 (−1.47 to −0.04)	0.038 *	−0.41 (−1.32 to 0.49)	0.360
Side-bending to left	31.34 (29.48 to 33.19)	31.53 (29.59 to 33.47)	32.31 (30.34 to 34.29)	0.19 (−0.46 to 0.85)	0.555	0.97 (0.26 to 1.68)	0.008 *
Rotating to right	50.80 (48.16 to 53.44)	50.56 (47.96 to 53.16)	48.19 (45.62 to 50.76)	−0.24 (−0.98 to 0.49)	0.510	−2.60 (−5.95 to 0.73)	0.122
Rotating to left	47.82 (45.51 to 50.14)	48.90 (46.43 to 51.37)	48.19 (45.62 to 50.76)	1.07 (0.18 to 1.96)	0.019 *	0.36 (−0.36 to 1.09)	0.314

Note: * indicates *p* < 0.05.

**Table 2:** Intra-rater and inter-rater reliability of craniocervical ranges measured by the hybrid device.

	Intra-Rater ICC (95% CI)	Intra-Rater SEM (Degrees)	Intra-Rater MDC (Degrees)	Inter-Rater ICC (95% CI)	Inter-Rater SEM (Degrees)	Inter-Rater MDC (Degrees)
Flexion	0.963 (0.933 to 0.980)	2.68	7.42	0.979 (0.962 to 0.989)	1.81	5.01
Extension	0.954 (0.916 to 0.975)	2.50	6.92	0.953 (0.914 to 0.975)	2.61	7.24
Side-bending to right	0.925 (0.864 to 0.959)	1.60	4.44	0.880 (0.786 to 0.934)	2.03	5.63
Side-bending to left	0.939 (0.888 to 0.967)	1.48	4.12	0.931 (0.875 to 0.963)	1.59	4.39
Rotating to right	0.959 (0.925 to 0.978)	1.66	4.61	0.942 (0.895 to 0.969)	1.96	5.44
Rotating to left	0.931 (0.874 to 0.962)	1.99	5.52	0.956 (0.919 to 0.976)	1.63	4.51

Note: CI, confidence interval; ICC, intraclass correlation coefficient (analyzed by the two-way random effect model); SEM, standard error of measurement; MDC, minimal detectable change.

**Table 3:** Comparisons between the hybrid device and universal goniometer for measurement of craniocervical ranges.

	First Rater First Evaluation in Degrees by the Hybrid Device (95% CI)	First Rater First Evaluation in Degrees by the Universal Goniometer (95% CI)	Between-Device Mean Difference in Degrees (95% CI)	*p* Value	Correlation Coefficient *r* Value (95% CI)	*p* Value	Coefficient of Repeatability (Degrees)
Flexion	41.80 (37.70 to 45.90)	41.58 (37.47 to 45.88)	0.12 (−0.92 to 1.16)	0.815	0.968 (0.941 to 0.983)	<0.001 *	6.428
Extension	51.48 (47.56 to 55.40)	51.58 (47.67 to 55.49)	−0.09 (−1.05 to 0.86)	0.838	0.969 (0.944 to 0.984)	<0.001 *	5.888
Side-bending to Right	34.51 (32.78 to 36.23)	34.53 (32.51 to 32.56)	−0.02 (−0.73 to 0.68)	0.944	0.941 (0.892 to 0.968)	<0.001 *	4.399
Side-bending to Left	31.34 (29.48 to 33.19)	30.58 (28.73 to 32.43)	0.75 (0.00 to 1.50)	0.047 *	0.918 (0.852 to 0.956)	<0.001 *	4.810
Rotating to Right	50.80 (48.16 to 53.44)	52.17 (49.52 to 54.81)	−1.36 (−2.37 to −0.36)	0.009 *	0.927 (0.867 to 0.961)	<0.001 *	6.720
Rotating to Left	47.82 (45.51 to 50.14)	49.51 (47.32 to 51.70)	−1.68 (−2.58 to −0.78)	0.000 *	0.921 (0.857 to 0.957)	<0.001 *	6.428

Note: * indicates *p* < 0.05.
